# Long-term Outcome of Arterial Stroke in Children

**DOI:** 10.15844/pedneurbriefs-29-5-4

**Published:** 2015-05

**Authors:** J. Gordon Millichap

**Affiliations:** 1 Division of Neurology, Ann & Robert H. Lurie Children's Hospital of Chicago, Chicago, IL; 2 Departments of Pediatrics and Neurology, Northwestern University Feinberg School of Medicine, Chicago, IL

**Keywords:** Pediatrics, Stroke, Outcomes

## Abstract

Investigators at University Children's Hospital, Inselspital, and Universities of Bern, Geneva, Basel, and Zurich, Switzerland compared long-term outcome of children (1 month-16 years) and young adults (16.1-45 years) with arterial ischemic stroke (AIS) using prospective data from the Swiss Neuropediatric Stroke Registry and the Adult Bernese stroke registry, between Jan 2000 and Dec 2008.

Investigators at University Children's Hospital, Inselspital, and Universities of Bern, Geneva, Basel, and Zurich, Switzerland compared long-term outcome of children (1 month-16 years) and young adults (16.1-45 years) with arterial ischemic stroke (AIS) using prospective data from the Swiss Neuropediatric Stroke Registry and the Adult Bernese stroke registry, between Jan 2000 and Dec 2008. Follow-up information was available in 95/116 children and 154/187 young adults. Median follow-up was 6.9 years (range 4.7-9.4). Long-term functional outcome was similar; 53 (56%) children and 84 (55%) young adults had a favorable outcome. Mortality in children was 14% and in young adults 7% (p=0.121), and recurrence rate did not differ (p=0.759). Except for more behavioral problems among children (13% vs 5%, p=0.040) and more effects of AIS on everyday life in adults (27% vs 64%, p<0.001), overall psychosocial impairment and quality of life did not differ. Low Pediatric NIH Stroke Scale/NIH Stroke Scale score was the most important predictor of favorable outcome (p<0.001). No major differences in mortality, disability, quality of life, psychological, or social variables were found in long-term outcome after AIS in children and young adults. [1]

COMMENTARY. Do children recover better from AIS than young adults? A question posed in an editorial, given the commonly held opinion among neurologists of a greater plasticity of the child's brain [2]. While commending the authors for the multiple strengths of the long-term study, they point to different interpretations of the findings. The plasticity in children might be more apparent if children are compared with older adults. The groups varied in terms of stroke etiology, a factor that may influence outcome. In the majority of patients the severity of residual symptoms did not change significantly within the year prior to follow-up. However, 23% of children and 14% of young adults reported ongoing improvements. While the study lends support to the concept of neuroplasticity, the results also introduce doubts that can be answered only by further long-term outcome studies.

Seizures are reported in 12 (15%) of 82 children and 15 (11%) of 139 young adults (p=0.403); active seizures in 1 (8%) and 1 (7%), (p=1.0). Poststroke seizures are of 2 types, early-onset (<14 days after the stroke) and late-onset (>14 days after). In a Taiwan study of 78 survivors of AIS in children aged 1 month to 18 years, 25% had early-onset seizures, and 13 (65%) of 20 survivors of early-onset seizures had late-onset seizures after the acute stage, of which 12 had post-stroke epilepsy [3]. A comparison of epilepsy following AIS in children and young adults would require records of the types of seizures and EEG.
